# Effects of clove oil concentrations on blood chemistry and stress-related gene expression in Siamese fighting fish (*Betta splendens*) during transportation

**DOI:** 10.3389/fvets.2024.1392413

**Published:** 2024-05-22

**Authors:** Chanoknan Sintuprom, Wirawan Nuchchanart, Sahabhop Dokkaew, Chak Aranyakanont, Raveewan Ploypan, Andrew P. Shinn, Ratchakorn Wongwaradechkul, Nguyen Dinh-Hung, Ha Thanh Dong, Satid Chatchaiphan

**Affiliations:** ^1^Department of Aquaculture, Faculty of Fisheries, Kasetsart University, Bangkok, Thailand; ^2^Department of Animal Science, Faculty of Agriculture at Kamphaeng Saen, Kasetsart University, Nakhon Pathom, Thailand; ^3^Department of Pathology, Faculty of Veterinary Science, Kasetsart University, Bangkok, Thailand; ^4^INVE Aquaculture, Nonthaburi, Thailand; ^5^Aquaculture Pathology Laboratory, School of Animal and Comparative Biomedical Sciences, The University of Arizona, Tucson, AZ, United States; ^6^Aquaculture and Aquatic Resources Management, Department of Food Agriculture and Bioresources, School of Environment, Resources and Development, Asian Institute of Technology, Pathum Thani, Thailand

**Keywords:** *Betta splendens*, transportation, stress, clove oil, animal welfare

## Abstract

Siamese fishing fish (*Betta splendens*) or betta are usually subjected to a special method of transportation for global trade, where they are individually conveyed in plastic bags containing just enough water to cover their bodies. This study aimed to investigate the effects of transportation on their stress response by measuring hematological values, stress hormone levels, glucose levels, and stress-related gene expression. Betta fish (average body weight 1.91 ± 0.42 g; *n* = 30) were exposed to simulated transport in a water volume of 40 mL for 12, 24, and 48 h. Baseline levels (pre-transport) were measured prior to the experiment. The control group was transported using water without adding clove oil. Two treatment groups were transported using water with the addition of 1 and 3 mg/L concentrations of clove oil, respectively. The results revealed that transportation can be a factor that affects water quality. The pH and dissolved oxygen levels were significantly lower than baseline, while nitrite and total ammonia concentrations significantly increased. Correlating to the stress responses, significantly increasing total red blood cell counts, plasma cortisol levels, and up-regulating the expression of stress-related genes, including *HSP70*, *GR*, *MR*, and *HIF-1α*. The addition of 1 mg/L clove oil was found to reduce stress during the transport simulation, as evidenced by a reduction in these stress parameters. Conversely, increasing the concentration of clove oil to 3 mg/L significantly increased plasma cortisol after 12 h of simulated transport, and up-regulated *GR*, *MR*, and *HIF-1α* expression. This study revealed that the transport process can stimulates stress in betta fish but adding a concentration of 1 mg/L clove oil to the transport water could mitigate this stress response and promote animal welfare during their transportation.

## 1 Introduction

Transportation is recognized as a significant source of stress resulting in measurable responses (1, 2). This stress is attributed to various risks arising from inadequate inappropriate stocking densities (3) vibration, potential manhandling, temperature changes (4), and deterioration in water quality (5). Such conditions can render fish susceptible to injuries (6) or can result in mortality during transit. Siamese fighting fish (*Betta splendens*), commonly known as betta, holds economic significance in the global ornamental fish trade. Male bettas are characterized by their aggressive behavior (7, 8) and are therefore typically transported individually in small plastic bags containing approximately 40 mL of water, maintaining a water-to-air ratio of 1:3 (7). This unique species-specific transport procedure has raised concerns from the People for the Ethical Treatment of Animals (PETA) regarding potential stress and welfare for betta fish (9). Prior studies have explored transportation stress responses in various species such as the dog snapper, *Lutjanus jocu* [see (10)], channel catfish, *Ictalurus punctatus* [see (11)], meagre, *Argyrosomus regius* [see (12)], and in Nile tilapia, *Oreochromis niloticus* [see (13)]. These stresses experienced by fish are linked to physiological responses, particularly endocrine alterations that induce changes in metabolic aspects, such as glucose levels, their hematological profile, and cellular changes. These changes encompass, the expression of stress-related genes in fish, varying depending on the species (14–18). In betta fish, an increase in the expression of trypsin, which is potentially associated with stress-related physiological effects, has been observed during transportation in the lowest water volume (40 mL) (19). However, there is limited literature specifically addressing the transportation of betta fish, particularly in terms of direct stress assessment.

Minimizing stress during live-fish transport poses a considerable challenge (20). In recent years, considerable additive extracts have been studied and integrated into welfare improvement (21–23). Especially, the incorporation of clove oil into the water during transportation has been explored in various fish species, such as common carp, *Cyprinus carpio* [see (24)], meagre (25), betta fish (26), clown fish, *Amphiprion ocellaris* [see (27)], and the European catfish, *Silurus glanis* [see (28)]. Clove oil, being a natural anesthetic, is readily available, cost-effective, safe, and effective in stress alleviation (29–32). Its active ingredients are eugenol and iso-eugenol, which not only reduces fish mortality but also enables rapid recovery (31). Dosing is crucial and varies based on species, fish size (33) exposure time, and environmental conditions (31). The concentration of clove oil used in the betta fish trials adhered to the recommendations provided by Sintuprom et al. (34), suggesting that the addition of clove oil could potentially aid in controlling water quality within an appropriate range during transportation. However, concentrations of 5 and 10 mg/L resulted in higher stress levels, possibly indicating that these concentrations were too high. Consequently, this study utilized this information to determine the optimal concentration of clove oil.

This study investigated stress indicators in Siamese fighting fish during simulated transport, including cortisol levels, glucose levels, hematological parameters, and the expression of stress-related genes. Additionally, the study assessed the efficacy of clove oil at concentrations of 1 and 3 mg/L to identify the most practical solution for farmers in mitigating stress and enhancing the welfare of bettas during transportation.

## 2 Materials and methods

### 2.1 Experimental design and transport procedures

Adult mixed-sex betta fish (*B. splendens*) aged 5 months, with an average body weight of 1.91 ± 0.42 g and an average standard length of 4.08 ± 0.39 cm (*n* = 30), were sourced from a local farm in Nakhon Pathom province, Thailand. The fish were individually housed in 250 mL glass bottles containing 140 mL of water. A total of 825 apparently healthy bettas were subjected to a 24-h fasting period and re-examined before the study began to ensure that they showed no external injuries or clinical signs.

Clove oil, sourced from Sigma-Aldrich, Singapore, with an eugenol content of 85% w/w was used. The concentration of clove oil used for the trials followed the recommendation provided by Sintuprom et al. (34). The preparation involved mixing clove oil with 95% ethanol (C_₂_H_₅_OH) in a 1:9 ratio (26, 31), to produce final concentrations of 1 and 3 mg/L of clove oil. All stock solutions were prepared immediately before the experiment.

**Figure 1:**
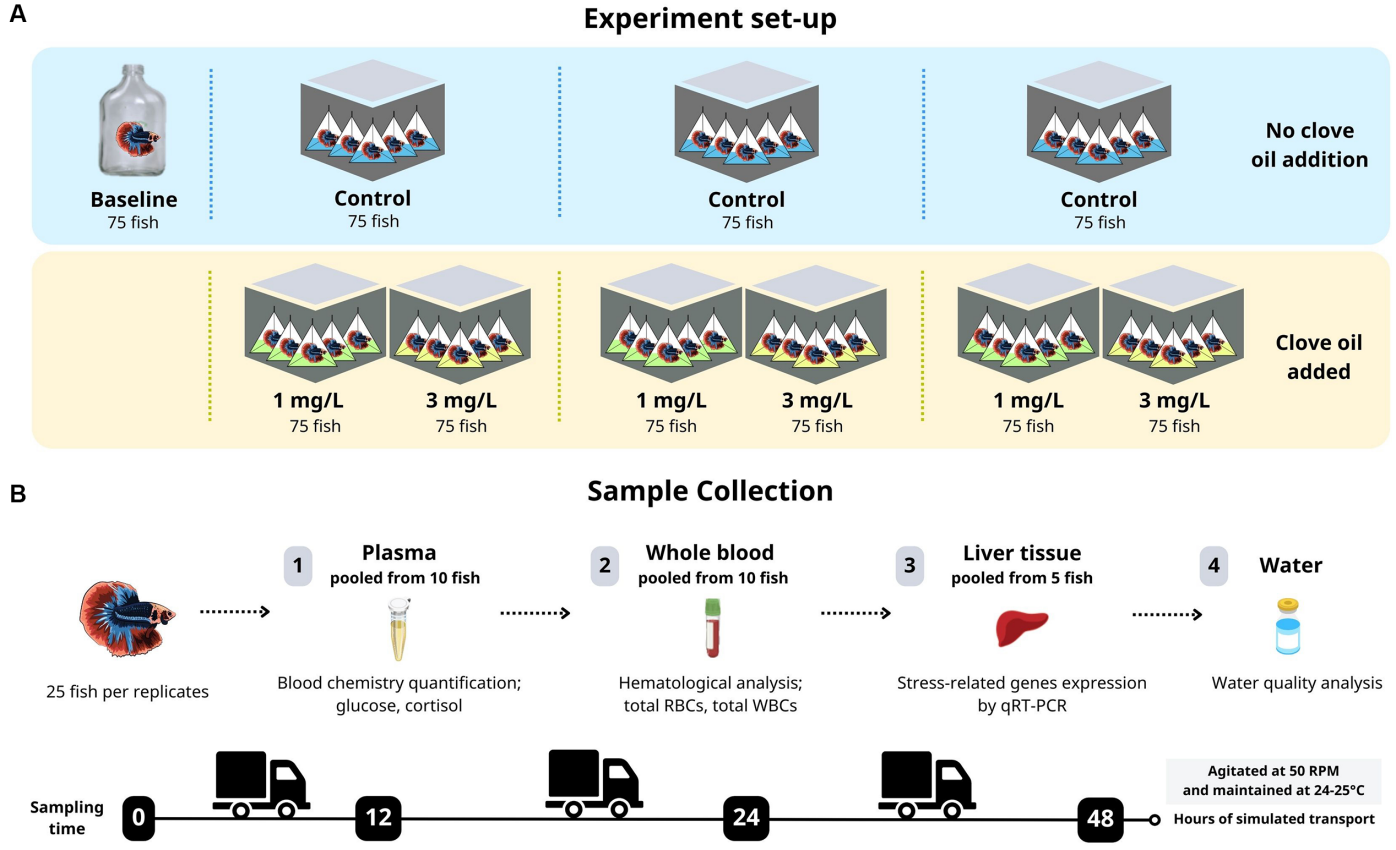
An overview of the experimental workflow implemented in this study. **(A)** Experiment set-up, **(B)** Sample collection.

The experiment was conducted with fish divided into four different groups. Before the experiment began, after a 24-h fasting period and just before the transport procedure, 75 fish were selected for the baseline-level group (pre-transport). These fish were organized into three replicates of 25 fish each. The remaining 750 fish were randomly assigned to one of three experimental groups: a control group without adding clove oil, and two treatment groups to which either 1 mg/L or 3 mg/L of clove oil was added. Each of the three experimental groups were further sub-divided and ran as three replicates. An overview of the experimental workflow in this study are illustrated in Figure 1.

Individual fish were placed in 3 × 5.5 inch plastic bags, containing 40 mL (i.e., one-third full) dechlorinated water with or without the respective amount of clove oil, following the methodology of Thongprajukaew et al. (19). The bags were then filled with pure oxygen and then sealed with an FRD 1,000 LW/S continuous sealer (Brother, China). These procedures align with established international transportation protocols (Dokkaew, personal communication, 2022) followed by betta farmers. The simulated transport procedures adhered to the methodology of Wu et al. (35). The pre-packaged fish were then placed in 11.8 × 16.9 × 11.4 inch styrofoam boxes with lids to protect them from light. Subsequently, they were subjected to an orbital shaker at 50 RPM for 48 h, and maintained at an internal temperature of 24°C–25°C. Samples of fish were then randomly collected from each of the three post-transport groups after 12, 24, and 48 h of simulated transport. The survival rate of the fish in each group was determined at each time point.

Prior to sample collection, the fish were euthanized promptly using 0.1% clove oil for 1–2 min that minimizes the stress response, following the procedure described by Davis et al. (36). The experimental procedures and the utilization of animals received approval from the Institutional Animal Care and Use Committee of Kasetsart University (approval ID: ACKU63-FIS-008).

### 2.2 Water quality measurement

Water quality was examined at each sampling point throughout the experiment. pH values were determined using an Ecoscan pH5/6 device (Eutech, United States). Temperature and dissolved oxygen (DO) levels were directly measured using an EcoSense DO 200-4 M instrument (YSI, Germany). Alkalinity was determined through a titration method (37), while nitrite (NO_2_^−^) concentration was assessed using a colorimetric method (37). Additionally, the total ammonia nitrogen (TAN) concentration was determined using Koroleff’s indophenol blue method (37).

### 2.3 Blood parameters

Blood samples from the fish processed at 0, 12, 24, and 48 h were assessed using two different methods for hematological analysis (i.e., total red/white blood cell counts) and blood chemistry quantification (i.e., concentration of plasma cortisol and plasma glucose levels). The procedures are outlined below.

For the hematological analysis, whole blood was collected using a heparinized capillary tube following tail ablation. Each sample of whole blood consisted of a pool of whole blood collected 10 fish. Three replicated were prepared. The whole blood pool sample was then divided and mixed with Natt and Herrick’s solution (at a ratio of 1:200) to allow for the subsequent determination of representative total RBC and WBC counts. Blood counts were determined from replicate counts made using a hemocytometer under an Olympus CX22 compound microscope (magnification 40X).

For blood chemistry analysis, the plasma collection method was adapted from Babaei et al. (38) with a modification involving the use of a perforated 0.5 mL tube inserted into a 1.5 mL standard microcentrifuge tube. Plasma was collected from a pool of 10 fish per replicate; the three replicates were then centrifuged at 400 × g for 5 min at 12°C. The samples were subjected to further centrifugation at 4,000 × g for 15 min at 12°C to separate the blood cells. The plasma was then collected and stored at −20°C until the cortisol and glucose content of each sample was determined. A volume of 40 μL of plasma from each replicate was used for the quantification of glucose using a GLU VT assay kit (IDEXX Laboratories, United States) with the VetTest Chemistry Analyzer (IDEXX Laboratories, United States). An additional aliquot of 40 μL from each replicate was taken to determine plasma cortisol levels using the General Cortisol ELISA kit (MyBioSource, United States), with measurements conducted within the range of 0.5–300 ng/mL. The optical density (OD value) of the samples thereafter was then determined at 490 nm using a BK-EL10C ELISA microplate reader (Biobase, China).

### 2.4 Stress-related genes expression by qRT-PCR

For this component of the study, livers pooled from five fish per replicate were used for analysis. These samples were promptly flash-frozen in liquid nitrogen and stored at −80°C until total RNA extraction. Total RNA extractions were conducted following the manufacturer’s protocols, utilizing TRIzol reagent (Thermo Fisher Scientific, United States). RNA samples were then processed using the GeneJET RNA Cleanup and Concentration Micro Kit (Thermo Fisher Scientific, United States), following the Total RNA Clean-up protocol. Qualitative and quantitative assessments of RNA were performed using a NanoDrop Eight Spectrophotometer (Thermo Fisher Scientific, United States).

To synthesize complementary DNA (cDNA), 1 μg of total RNA per sample was used with the RevertAid First Strand cDNA Synthesis kit (Thermo Fisher Scientific, United States). Primer sequences used for the qRT-PCR analyses are listed in Table 1. The study investigated transcript levels of heat shock protein (*HSP70*), heat shock protein (*HSP90*), glucocorticoid receptor (*GR*), mineralocorticoid receptor (*MR*), and hypoxia-inducible factor 1-alpha (*HIF-1α*), by employing *β-actin* as a housekeeping gene.

**Table 1:** Primer sequences used for the gene expression analysis in Siamese fighting fish (*Betta splendens*).

Genes	Function	Sequence (5′–3′)		Length (bp)	References/accession no.
*β-actin*	House-keeping gene	*F*	AGGCTGTGCTGTCCCTGTAT	200	Amparyup et al. (40)
		*R*	GAAGGAGTAGCCACGCTCTG		
*HSP70*	Protein folding and repair/degradation	*F*	GGGAGCTGAACAAGAGCATC	190	This study/XM_029157549.2
		*R*	ATGGTGGTGTTCCGTTTGAT		
*HSP90*	Steroid hormone receptors	*F*	CAAGAACGACAAGGCTGTGA	193	This study/XM_029141667.2
		*R*	AATTTCGTCGGGAACAGATG		
*GR*	Corticosteroid receptors	*F*	GAACTGGCAGCGCTTTTATC	197	This study/XM_029164787.2
		*R*	GGTGGAACAGGAGAGCTTTG		
*MR*	Corticosteroid receptors	*F*	AGCGCAAGGAACAATGTCTT	191	This study/XM_029149835.2
		*R*	AGCTGGACATGCTGTCTGTG		
*HIF-1α*	Hypoxia transcriptional regulator	*F*	TGGGCTATGATCCAGAGGAC	200	This study/XM_029138456.1
		*R*	AGTTTCCACCCACACAAAGC		

*β-actin*; beta-actin, *HSP70*; heat shock protein 70, *HSP90*; heat shock protein 90, *GR*; glucocorticoid receptor, *MR*; mineralocorticoid receptor, *HIF-1α*; hypoxia-inducible factor 1-alpha.

qRT-PCR reactions were prepared in 25 μL volumes, comprising 12.5 μL of 2X SYBR Green QPCR master mix and 10 μM of each primer. The amplification conditions were as follows: an initial pre-denaturation at 94°C for 2 min, followed by 40 cycles of denaturation at 98°C for 10 s, annealing at 55°C for 30 s, extension at 68°C for 10 s, and a final extension at 68°C for 10 s. These reactions were carried out utilizing the Mastercycler ep realplex Real-time PCR System (Eppendorf, Germany). Relative gene expression analysis was performed using the 2^−ΔΔ CT^ method, as described by Livak and Schmittgen (39).

### 2.5 Statistical analysis

All data analyses were performed using IBM SPSS Statistics 26. One-way analysis of variance (ANOVA) tests were used to examine differences between the three groups at each time interval and differences within each group across various time intervals, followed by Duncan’s *post-hoc* test for pairwise comparisons. Statistical significance was set at a threshold of *p* < 0.05.

## 3 Results

### 3.1 Survival rate and water quality parameters

The survival rate (%) of the fish following exposure to the simulated transport conditions was determined at the end of the trial and calculated from a total of 250 fish per group. The addition of 1 mg/L clove oil resulted in the highest survival rate of 98.4%, followed by 97.6% in the control group, and 96.8% in the group receiving 3 mg/L clove oil.

**Figure 2:**
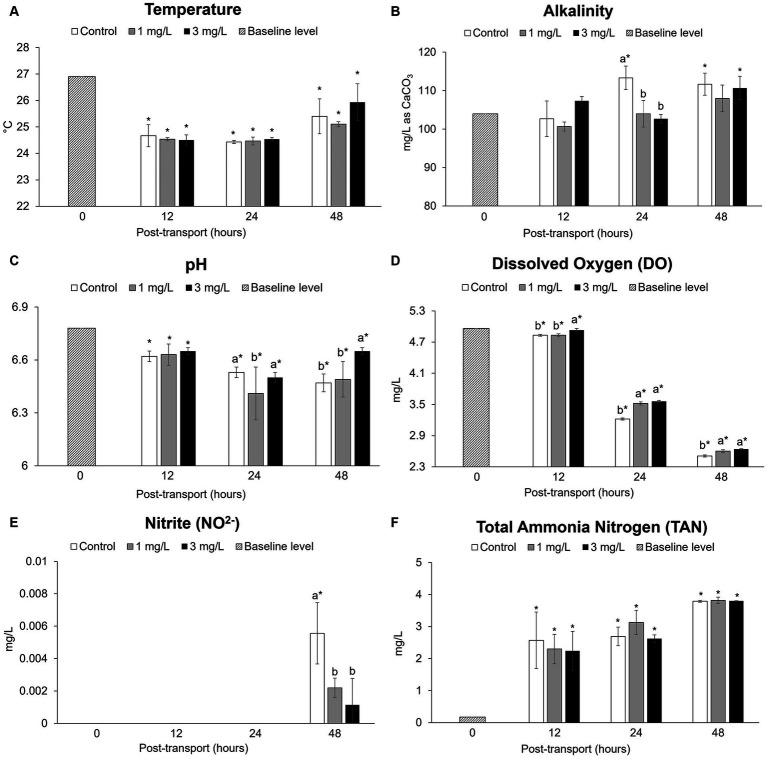
Water quality parameters during the *Betta splendens* (*n* = 3) transportation simulation. **(A)** Temperature, **(B)** Alkalinity, **(C)** pH, **(D)** Dissolved oxygen, **(E)** Nitrite, **(F)** Total ammonia nitrogen value. Vertical bars indicate standard deviation; mean with different lowercase letters indicate significant differences (*p* < 0.05) among treatments at each time interval, according to one way-ANOVA. Asterisks indicate significant differences (*p* < 0.05) between the groups and the baseline level, according to the ANOVA.

The mean values of water temperature, alkalinity, pH, DO, nitrite, and TAN are presented in Figure 2. In the post-transport groups, the average temperature was 24.84 ± 0.59°C (Figure 2A). Alkalinity showed significant differences compared to the baseline level (*p* < 0.05) observed in the control group and in the group of fish receiving 3 mg/L clove oil after 24 h of simulated transport (Figure 2B). The pH values (Figure 2C) and in DO levels (Figure 2D) decreased significantly in post-transport groups when compared to the baseline level (*p* < 0.05); these parameters gradually decreased with increasing transportation time. Nitrite concentrations (Figure 2E) showed significant differences (*p* < 0.05) after 48 h of simulated transport. TAN concentrations (Figure 2F) showed a significant increase in post-transport groups (*p* < 0.05), with the highest recorded value at 48 h of simulated transport.

### 3.2 Hematological values

**Figure 3:**
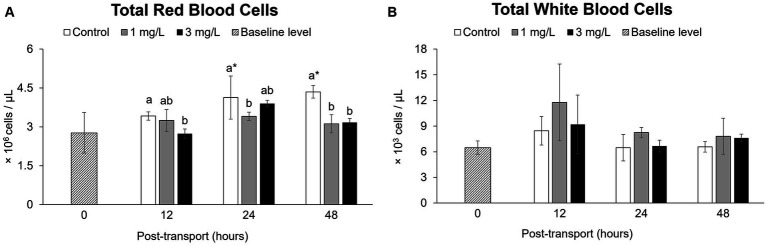
Hematological parameters during the *Betta splendens* (*n* = 3) transportation simulation. **(A)** Total red blood cells, **(B)** Total white blood cells. Vertical bars indicate standard deviation; mean with different lowercase letters indicate significant differences (*p* < 0.05) among treatments at each time interval, according to one-way ANOVA. Asterisks indicate significant differences (*p* < 0.05) between the groups and the baseline level, according to the ANOVA.

The values for the control group had increased significantly (*p* < 0.05), reaching the highest total RBC count at 48 h (*p* < 0.05) after the start of the simulated transport. In a contrast to this, the treatment groups receiving 1 mg/L clove oil had significantly lower total RBC counts (*p* < 0.05), with no significant differences when compared to the baseline level (*p* > 0.05) throughout the experiment (Figure 3A).

No significant differences were noted when comparing these total WBC counts with those obtained from the control and two treatment groups at each time interval (*p* > 0.05) by the end of the trial (*p* > 0.05; Figure 3B).

### 3.3 Stress hormone levels

**Figure 4:**
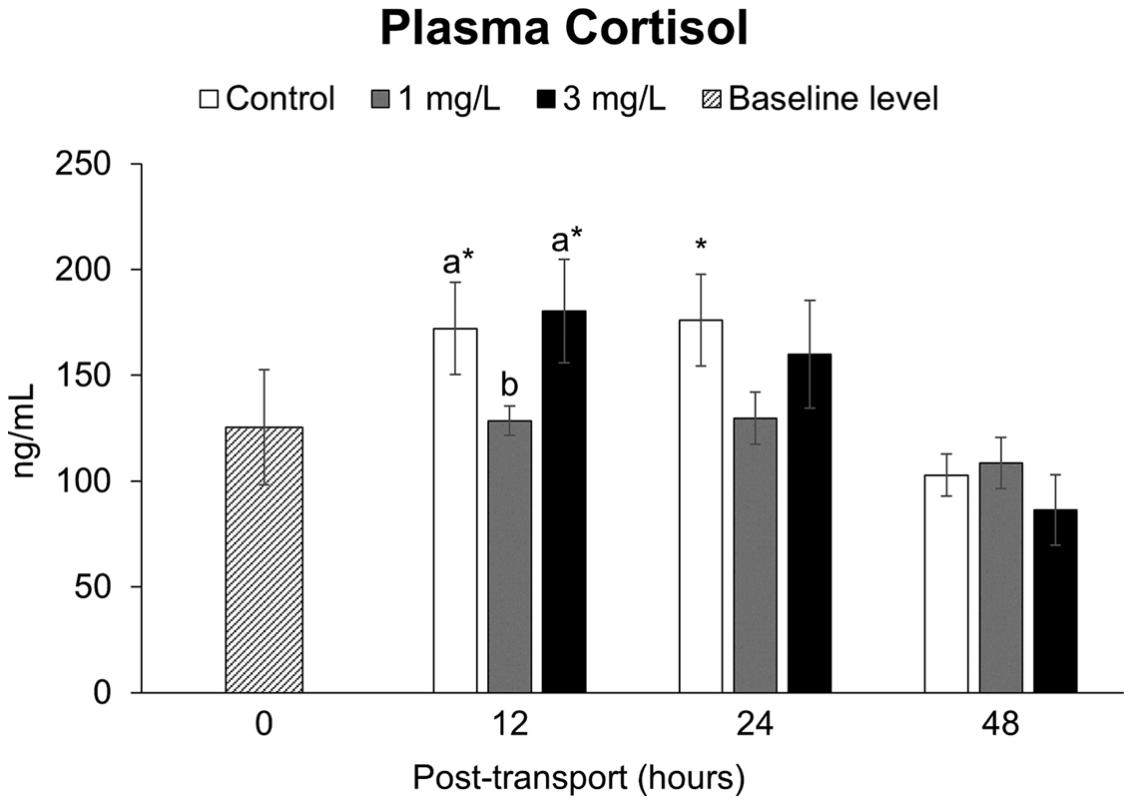
Stress hormone levels (plasma cortisol levels) during the *Betta splendens* (*n* = 3) transportation simulation. Vertical bars indicate standard deviation; mean with different lowercase letters indicate significant differences (*p* < 0.05) among treatments at each time interval, according to one-way ANOVA. Asterisks indicate significant differences (*p* < 0.05) between the groups and the baseline level, according to the ANOVA.

The concentrations of plasma cortisol (Figure 4) in the control and in the treatment group receiving 3 mg/L clove oil after 12 h of simulated transport were significantly higher (*p* < 0.05) than the baseline level. In contrast, the treatment group receiving 1 mg/L clove oil exhibited a significantly lower (*p* < 0.05) level of plasma cortisol compared to the other two groups, with no statistically significant differences compared to the baseline level (*p* > 0.05). After 24 h, the cortisol levels in the control group exhibited a significantly elevated plasma cortisol concentration when compared to the baseline group (*p* < 0.05). There were no significant differences (*p* > 0.05) between the control and the two treatment groups at any other time point, and they remained comparable to the baseline (*p* > 0.05) at 48 h.

### 3.4 Plasma glucose levels

**Figure 5:**
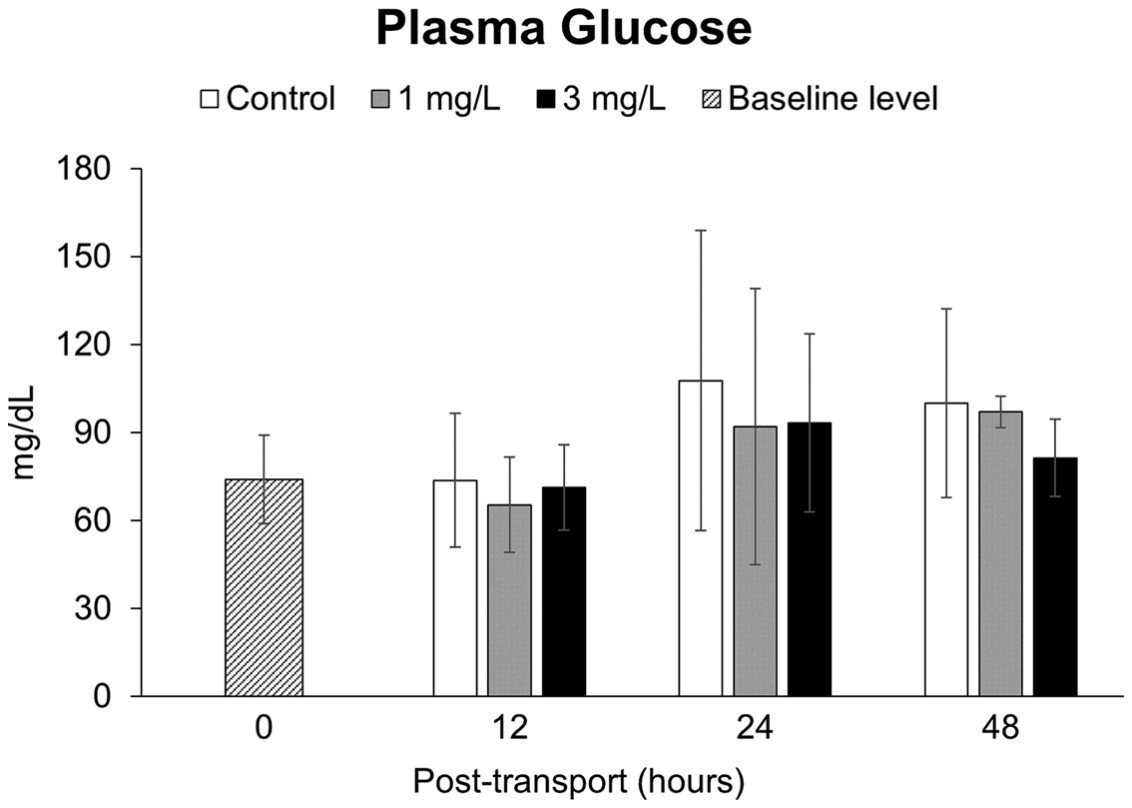
Plasma glucose levels during the *Betta splendens* (*n* = 3) transportation simulation. Vertical bars indicate standard deviation; mean with different lowercase letters indicate significant differences (*p* < 0.05) among treatments at each time interval, according to one-way ANOVA. Asterisks indicate significant differences (*p* < 0.05) between the groups and the baseline level, according to the ANOVA.

There were no statistically significant differences in the concentration of plasma glucose (Figure 5) between the control and two treatment groups, as well as between three groups and the baseline level, over the 48 h under simulated transport (*p* > 0.05).

### 3.5 Stress-related gene expression

**Figure 6:**
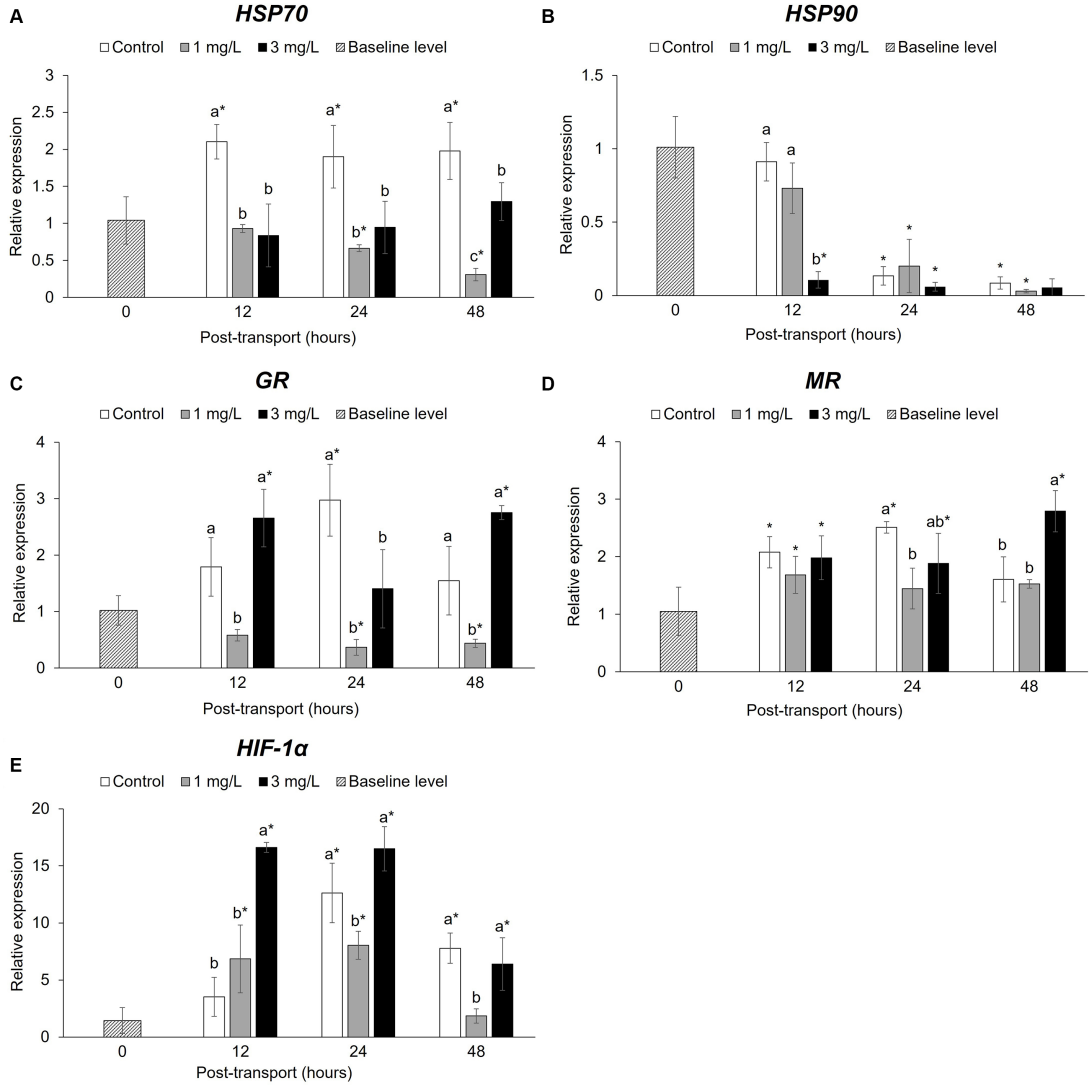
Stress-related gene expression during the *Betta splendens* (*n* = 3) transportation simulation. **(A)** *HSP70*, **(B)** *HSP90*, **(C)** *GR*, **(D)** *MR*, **(E)** *HIF-1α*. Vertical bars indicate standard deviation; mean with different lowercase letters indicate significant differences (*p* < 0.05) among treatments at each time interval, according to one-way ANOVA. Asterisks indicate significant differences (*p* < 0.05) between the groups and the baseline level, according to the ANOVA.

There was a significant difference (*p* < 0.05) in the relative expression of *HSP70* (Figure 6A) between the control and the two treatment groups following the transport simulation. After 12 h, levels in the control group had significantly increased (*p* < 0.05) when compared to the baseline level, this remained significantly higher than the other tests groups. Conversely, the two treatment groups were significantly lower (*p* < 0.05) than the control throughout the experiment. The addition of 1 mg/L clove oil resulted in a significant down-regulation (*p* < 0.05) in *HSP70* expression and significant differences when compared to the baseline level at 24 and 48 h.

The study also found significant differences in the levels of *HSP90* (Figure 6B) between the control and the two treatment groups. At 12 h, the addition of 3 mg/L clove oil demonstrated a significant reduction (*p* < 0.05) in *HSP90* expression compared to the baseline level; this was significantly lower (*p* < 0.05) than the other groups. After 24 and 48 h of simulated transport, the levels of *HSP90* in all post-transport groups had significantly decreased (*p* < 0.05), with no significant differences (*p* > 0.05) between the control and the two treatment groups.

After 12 h simulated transport, *GR* levels (Figure 6C) had significantly increased (*p* < 0.05) in the control and 3 mg/L clove oil groups. The addition of 1 mg/L clove oil led to a significant decrease (*p* < 0.05) in *GR* expression, which was lower than the other groups, including the baseline level, at 24 and 48 h. While the control group was not significantly different (*p* > 0.05) from the baseline level, the addition of 3 mg/L clove oil resulted in a significant increase (*p* < 0.05) in the *GR* expression of the fish evaluated at 48 h.

The expression of *MR* (Figure 6D) was found to have significantly increased (*p* < 0.05) in all post-transport groups at 12 h compared to the baseline level. By 24 h, however, expression levels in the fish receiving the 1 mg/L clove oil treatment significantly decreased (*p* < 0.05). The control and other treatment groups also displayed variations in expression, notably in the group receiving 3 mg/L clove oil, which were significantly higher when compared to the baseline throughout the experiment. In contrast, slight down regulation of *GR* was observed in fish that received 1 mg/L clove oil at 24 and 48 h.

There were also significant increases (*p* < 0.05) in the expression levels of *HIF-1α* (Figure 6E) in the two treatment groups at 12 h. After 24 h, all post-transport groups showed a significant difference (*p* < 0.05) in *HIF-1α* when compared with the baseline. The 1 mg/L clove oil group had a significantly lower (*p* < 0.05) level of expression than those in the control and the 3 mg/L clove oil groups. At 48 h, *HIF-1α* expression in the fish receiving the 1 mg/L clove oil treatment a decreased and this was not significantly different from the baseline level.

## 4 Discussion

The burgeoning ornamental fish industry necessitates the transportation of live fish. Ornamental fish, with their unique characteristics, often endure long-distance travel in sealed plastic bags (41). Water quality deterioration is a predictable adverse effect of transportation (5). This is one of the most important triggers that can lead to stress (42). The observed decrease in dissolved oxygen in this study suggests an increase in carbon dioxide, a consequence of fish respiration, which can result in a sequential reduction in pH as was seen in the control group. The concentration of nitrite in each group was below detectable levels for much of the trial, however, levels were found to have increased after 48 h with an increase in fish metabolite accumulation (6). The increase in total ammonia nitrogen concentrations indicates metabolic waste accumulation, although the addition of 1 and 3 mg/L clove oil during transport showed no significant influence on reducing the amount of ammonia produced. Following the recommendations for betta transport, maintaining a constant water temperature during transport has advantages as it affects the solubility of oxygen and decreases ammonia toxicity (20). Therefore, even the calculated highest record of unionized ammonia (NH_3_-N) was 0.0081 mg/L, which are within the threshold for general aquaculture production (43). According to Hong et al. (44), a study involving golden pompano (*Trachinotus ovatus*) found that pH values dropped and the concentration of high unionized ammonia increased after 8 h of simulated transport; the trial concluded that most of the physiological and biochemical indicators that were measured were not significantly affected. This indicates adverse changes in pH, ammonia, or nitrite are considered a common issue that occurs with the transportation process (11, 19, 44) and within an acceptable threshold, according to Laongsiriwong (43).

Clove oil has been shown to slow respiration rates and oxygen consumption (30, 45), as evidenced by higher dissolved oxygen levels in the groups receiving the 1 and 3 mg/L clove oil treatments than in the control group. Furthermore, nitrite concentrations were significantly lower than in the control group of this study due to the slowdown of metabolic excretion.

Transport can provoke an increase in the number of total RBCs. This has been reported several studies investigating levels in a range of fish species, including common carp (46) and in dog snapper *Lutjanus jocu* [see (10)]. Transport generated stress on betta fish in the current study, resulted in a marked increase in total RBC counts after 12 h of simulated transport. Coping with stress in the first period requires high energy demand, which includes mobilizing the number of RBCs. This response is essential, allowing the fish maximize the oxygen transport capabilities of its hemoglobin to serve increased energy demand (47, 48). Adding clove oil at a concentration of 1 and 3 mg/L, however, has the potential to reduce total RBC counts throughout the 48-h transport simulation when compared to the control without clove oil. This result follows the properties of anesthetics, which reduce metabolic activity and oxygen consumption (20, 31). Short-term stress sometimes results in an increase in total WBCs (49). According to Jia et al. (50), clove oil has antioxidant properties that can improve the immune system and has been shown to increase WBC number or increase phagocytic activity during bacterial challenge (51). This was not observed in the current study, in which clove oil did not significantly alter the total WBC counts during betta fish transport. The findings differ from previous research on short-term stress, such as handling stress or infected conditions mentioned above.

Stress is an energy-consuming process in which the fish’s perception of a stressor triggers three main cascade responses (52). The primary reaction is a neuroendocrine response that involve an increased release of corticosteroid hormone. Cortisol levels often rise during transportation (42), which boosts metabolism as an immediate energy source for fight or flight responses (14, 53). These observations have been documented in studies with several fish species, including golden pompano, *Trachinotus ovatus* [see (44)], rohu, *Labeo rohita* [see (54)], blood parrot cichlid, *Amphilophus citrinellus* × *Cichlasoma synspilum*, and koi, *C. carpio* [see (35)]. The betta fish transportation approach used in the current study induced plasma cortisol for a brief period of 12 h, which then reverted to baseline levels throughout the remainder of the trial (i.e., as evaluated at the 24 and 48 post-start time points). This aligns with prior research that found suitable transportation conditions ensure adequate density and water quality are within acceptable criteria; thereafter, stress-related parameters can spontaneously return to normal within 24 h (11, 55, 56).

Furthermore, the addition of 1 mg/L clove oil can relieve stress, as indicated by the reduction of cortisol levels throughout the experiment when compared to the control group without clove oil. Clove oil is classified as a local anesthetic, which affects systemically by inhibiting the respiratory center in the medulla oblongata linked to central nervous system depression. In the context of this study, light clove oil doses (i.e., 1 mg/L) reduce transport stress by allowing fish to maintain equilibrium and reduce swimming activity (20, 31), resulting in serenity and reduced utilization of energy in stressful conditions. According to Akar (29), using 1 mg/L clove oil in blue tilapia, *Oreochromis aureus*, for 72 h during their transportation can reduce cortisol levels. In contrast to this, raising the concentration to 3 mg/L of clove oil can induce cortisol levels at the 12-h evaluation point like the findings of this study. Earlier studies have shown that using a high concentration of clove oil can induce the production of cortisol (29, 32, 57). Elevated levels may lead to hypoxia and acidosis due to a decrease in oxygen and an increase in carbon dioxide in the blood, resulting in stress or death (58, 59).

Elevated glucose levels are result of an increased catabolism through the gluconeogenesis and glycogenolysis pathways (14, 47). This increase is associated with a range of secondary responses that are modulated by hormones and circulate in the bloodstream to ensure immediate availability of energy. In the current study, no significant variations in plasma glucose were found, which is considered favorable signaling (60). This finding supports the opinion of Martínez-Porchas et al. (61) who stated that glucose should be used as a complement to stress assessments rather than being used as the main indication of stress.

Heat shock proteins (HSPs) mainly resolve or degrade damaged proteins under stressful circumstances (62). HSP70 is a major stress-induced protein in the HSP family (63) and quantifying it can be useful for determining the impact of a particular environment. HSP90, by comparison, influences cytoskeleton components and steroid hormone receptor regulation (64). Several studies have indicated that *HSP70* and *HSP90* expression can be utilized as cellular stress biomarkers for animals in transport (11). In the current study, the betta transport resulted in elevated *HSP70* expression, which was significantly increased in the control group throughout the trial. This finding aligns with previous studies on spotted knifejaw (*Oplegnathus punctatus*) [see (65)], common carp (*C. carpio*), rainbow trout (*Oncorhynchus mykiss*) [see (66)], and on European seabass (*Dicentrarchus labrax*) [see (67)]. This study suggests that the addition of clove oil at doses of 1 and 3 mg/L were able to inhibit *HSP70* expression throughout the transport process whereas in normal conditions without clove oil, the expression of *HSP70* would be triggered. The findings of the current study are consistent with those of Teles et al. (68), who conducted in a study using 42.7 ± 6.8 g gilthead seabream (*Sparus aurata*) found that a dose of 2.5 mg/L clove oil was able to reduce *HSP70* levels for 24 h during their transportation. In contrast, the addition of clove oil appeared to have no significant effect on the expression of *HSP90* under simulated transportation condition.

Cortisol mediates its effects by activating the glucocorticoid receptor (*GR*) and the mineralocorticoid receptor (*MR*), which stimulates gene expression for physiological changes (69, 70). The transport process employed for betta did not exhibit an increase in *GR* expression, while the expression of *MR* was found to have increased in all post-transport groups at the 12-h evaluation point. A study conducted by Bortoletti et al. (12) using meagre fish (*Argyrosomus regius*), found that transport did not result in an increase in *GR* levels in the liver. The results of the current study indicate that adding 1 mg/L clove oil can reduce *GR* and *MR* expression when compared to a control group that did not receive a dose of clove oil or to those fish which were transported in 3 mg/L clove oil. This finding suggests that low doses of clove oil when used for this species might have stress-relieving capabilities.

Betta fish are air-breathing anabantids that possess a suprabranchial organ that serves as a site of aerial respiration; this is also known as the labyrinth organ (71). The natural habitats of betta fish are frequently hypoxic and anoxic at the bottom, due to high water temperatures and high organic content (72). In their development, betta fish can at least partially escape hypoxia (72–75), and they may have a greater tolerance to aquatic hypoxia (7, 76). Hypoxia-inducible factors (*HIF*s), however, are critical regulators of the transcriptional response to hypoxic stress (77, 78). Previous research on *HIF-1α* in fish such as rainbow trout (50, 78, 79) and spotted knifejaw (77) has demonstrated its role in regulating oxygen adaptation and hypoxia tolerance (50, 77–79). In the current study, the transport process employed for betta exhibited an increase in *HIF-1α* expression, which returned to the baseline after 48 h of simulated transport in the treatment group that received a dose of 1 mg/L clove oil. The other experimental groups tended to show a decrease in expression. According to Da Silva et al. (80), most aquatic organisms appear to be well adapted to cope with periodic fluctuations in oxygen levels, influenced by various factors such as loading density, water temperature, and pure oxygen infusion. Similarly, Honryo et al. (81) discovered an increase in *HIF-1α* expression levels in the gills, as well as in whole-body cortisol levels, in juvenile Pacific bluefin tuna, *Thunnus orientalis*, exposed to transport stress immediately after their release into sea cage; the fish, however, were reported to recover within 24 h.

## 5 Conclusion

The investigation of stress indicators during the transportation of Siamese fighting fish has provided important insights. Transport variables were identified as potential sources of stress, as evidenced by elevated levels of total RBC and plasma cortisol, as well as upregulation of specific stress-related genes such as *HSP70*, *GR*, *MR* and *HIF-1α*. This highlights the physiological and cellular responses of fish to transportation compared to pre-transport fish, which have fewer stress indicators. However, such responses are common during fish transportation. It was found that the addition of 1 mg/L clove oil to the water reduced these stress indicators compared to the untreated control group. Conversely, increasing the clove oil concentration to 3 mg/L significantly increased some stress parameters, including plasma cortisol, *GR*, *MR* and *HIF-1α*. Consequently, these results suggest that the addition of clove oil at a concentration of 1 mg/L to the water is an appropriate strategy to reduce stress levels during short-term (12 h) or long-term (24–48 h) transportation of betta fish.

## Data availability statement

The original contributions presented in the study are included in the article/supplementary material, further inquiries can be directed to the corresponding author.

## Ethics statement

The animal study was approved by the Institutional Animal Care and Use Committee of Kasetsart University (approval ID: ACKU63-FIS-008). The study was conducted in accordance with the local legislation and institutional requirements.

## Author contributions

CS: Conceptualization, Formal analysis, Investigation, Methodology, Writing – original draft, Writing – review & editing, Validation. WN: Validation, Writing – original draft, Writing – review & editing. SD: Writing – original draft, Writing – review & editing, Resources. CA: Writing – original draft, Writing – review & editing, Validation. RP: Methodology, Resources, Validation, Writing – original draft, Writing – review & editing. AS: Validation, Writing – original draft, Writing – review & editing. RW: Validation, Writing – original draft, Writing – review & editing. ND-H: Validation, Writing – original draft, Writing – review & editing. HD: Validation, Writing – original draft, Writing – review & editing. SC: Conceptualization, Funding acquisition, Project administration, Resources, Supervision, Validation, Writing – original draft, Writing – review & editing.
